# Lipid alterations in acute myocardial infarction are associated with gut microbiota

**DOI:** 10.1128/spectrum.02370-24

**Published:** 2025-02-19

**Authors:** Jiebin Zuo, Panpan Wang, Kewen Xue, Yuwen Tan, Ting Zhang, Yang Li, Feixiang He, Weikun Wu, Zhixiang Yan, Li Cong, Gang Li

**Affiliations:** 1 Cardiac Surgery and Structural Heart Disease Unit of Cardiovascular Center, The Fifth Affiliated Hospital, Sun Yat-sen University, Zhuhai, China; 2 Guangdong Provincial Engineering Research Center of Molecular Imaging, The Fifth Affiliated Hospital, Sun Yat-sen University and Southern Marine Science and Engineering Guangdong Laboratory (Zhuhai), Zhuhai, China; 3 Department of Endocrinology and Metabolism, The Fifth Affiliated Hospital, Sun Yat-sen University, Zhuhai, China; 4 Guangdong–Hong Kong–Macao University Joint Laboratory of Interventional Medicine, The Fifth Affiliated Hospital, Sun Yat-sen University, Zhuhai, China; U.S. Food and Drug Administration, Jefferson, Arkansas, USA

**Keywords:** acute myocardial infarction, lipidomics, proteomics, gut microbes

## Abstract

Although lipid metabolism and gut microbiota dysregulation are known to participate in cardiovascular disease, few studies have examined these alterations and associations in acute myocardial infarction (AMI) patients. This study reveals altered and associated lipid metabolism and gut microbiome based on proteomic and lipidomic analyses of fecal and plasma samples from 30 patients with AMI and 33 healthy volunteers. Twenty-three differential plasma lipids, nine altered fecal lipids, and nine differential fecal bacterial species were identified in AMI patients relative to healthy volunteers, with nine plasma lipids, three fecal lipids, and two fecal bacteria as potential biomarkers. Correlation analysis revealed that the abundances of *Stenotrophomonas* and *Bilophila* were positively correlated with sphingosine and ceramide levels, respectively. *Alistipes indistinctus* and *Porphyromonas* were correlated with blood lipid indicators (total cholesterol, triglycerides, and low-density lipoprotein-C), and *A. indistinctus*, *Bilophila*, and *Stenotrophomonas* were associated with myocardial injury indicators (cTnI, CK, CK-MB, LDH, and BNP). This study identified potential lipid and gut microbe biomarkers and their associations in AMI patients.

**IMPORTANCE:**

Acute myocardial infarction (AMI) remains a leading cause of morbidity and mortality worldwide. While lipid metabolism and gut microbiota are known to play important roles in cardiovascular diseases, their interactions in the context of AMI are not fully understood. In this study, we explore the lipidomic and microbiome alterations in AMI patients, identifying key biomarkers associated with myocardial injury. By correlating specific lipid changes with bacterial species in fecal samples, we highlight the potential of lipid–microbe interactions in the pathogenesis of AMI. These findings provide novel insights into the complex mechanisms underlying AMI and suggest potential targets for early diagnosis and therapeutic interventions aimed at modulating lipid and microbial profiles to improve patient outcomes.

## INTRODUCTION

Cardiovascular diseases are among the most significant global public health challenges, and acute myocardial infarction (AMI) is one of the most perilous. According to the Global Health Estimates 2019 report, the number of global deaths from coronary heart disease increased to nearly 9 million in 2019, accounting for 16% of all deaths (1). AMI is caused by coronary artery spasm or thrombosis, leading to myocardial ischemia, hypoxia, and necrosis. The pathogenesis of cardiovascular diseases is complex, and lipid metabolism disorders are important risk factors (2).

Lipids are among the most important biomolecules, playing critical roles in the structure and function of cell membranes (3, 4). Their roles in the progression of cardiovascular diseases have recently attracted attention. Lipid oxidation participates in the pathogenesis of cardiovascular diseases, primarily in those based on atherosclerosis. Oxidized lipids and their conjugates have been extensively characterized and are associated with several processes responsible for the pathogenesis and development of atherosclerosis, including endothelial cell dysfunction and inflammation (5, 6).

In recent years, with the continuous development of omics technologies, plasma lipidomics has been applied to the study of AMI. These studies have contributed to the identification of diagnostic biomarkers for AMI (7, 8). Meanwhile, lipid absorption and metabolism in the gut also affect plasma lipids (9). Fecal lipid profiles have been shown to differ between individuals with obesity (10). For instance, fecal lipidomics revealed 417 significantly elevated lipids in individuals with metabolic syndrome with diacylglycerol phosphocholine strongly correlated with metabolic syndrome risk factors (11).

In recent years, the impact of gut microbiota on cardiovascular health and disease has attracted great attention (12). Gut microbes interact with the host in various ways, including inflammatory responses, cholesterol metabolism, and bile acid synthesis (13–15). Specific gut microbes are involved in lipid metabolism and transformation, contributing to lipid homeostasis (16). For instance, *Bacteroides* and *Prevotella* in the gut can ferment dietary fiber to produce short-chain fatty acids (17). Gut microbiome composition can explain 4% of the variation in high-density lipoprotein cholesterol levels, 5% of the variation in body mass, and up to 6% of the variation in triglyceride levels (18). Individuals with high gut microbiome diversity typically have healthier blood lipid levels (19). In addition to the influence of the gut microbiota, host proteins in the gut also play crucial regulatory roles, such as in the selective degradation of gut microbial flora and recognition of metabolites produced by the gut flora (20). In this study, we studied the host–gut microbiota–lipid interaction in AMI by analyzing fecal and plasma lipids and gut microbiota via multi-omics technologies.

## MATERIALS AND METHODS

### Participant recruitment and sample collection

This study recruited 30 patients with AMI and 33 healthy control volunteers from July to October 2022 at the Fifth Affiliated Hospital of Sun Yat-sen University. Fecal and plasma samples were collected, along with clinical information during hospitalization. Clinical 01 included total cholesterol (TC) and low-density lipoprotein cholesterol (LDL-C); Clinical 02 comprised cardiac troponin I (cTnI), creatine kinase (CK), creatine kinase-MB (CK-MB), lactate dehydrogenase (LDH), and brain natriuretic peptide (BNP). This study was reviewed and approved by the Ethics Committee of the Fifth Affiliated Hospital of Sun Yat-sen University (K146-1). All participants and their families were informed about the purpose and process of the study. They voluntarily participated and cooperated in all aspects related to the study and signed written informed consent forms. Acute myocardial infarction: diagnosis was based on elevated necrotic myocardial serum biomarkers (cardiac troponin and myocardial enzyme levels), with at least one value above the 99th percentile upper reference limit and at least one of the following conditions: (1) ischemic chest pain; (2) new significant ST-T wave changes or new left bundle branch block; (3) appearance of pathological Q waves on an ECG; (4) new evidence of viable myocardium loss or new local wall motion abnormalities in imaging; (5) coronary artery thrombosis identified by angiography.

### Fecal and plasma sample collection

Fecal samples were collected in the first morning after the patient’s admission and placed in sterile fecal collection bags, labeled, and immediately transported to the laboratory on ice. The fecal sample was aliquoted to at least three pieces and placed into 5-mL cryotubes with each sample duplicated twice and then stored at −80°C. Plasma samples were collected in the first morning after admission from fasting participants. Blood was collected in sodium heparin anticoagulant tubes, and plasma separation was completed within an hour using a cold centrifuge at parameters (3000 rpm, 4°C, 10 minutes). The upper layer of plasma was then aliquoted into 1.5-mL cryotubes (0.5 mL/tube), labeled, and stored at −80°C. All samples were free from repeated freeze–thaw cycles.

### Lipid sample preparation

For fecal samples: 200 µL of pre-cooled chloroform: methanol (3:1) was added to 50 mg of the fecal sample, vortexed vigorously for 30 seconds, and then centrifuged at 16,000 *g* at 4°C for 15 minutes. The supernatant (100 µL) was transferred to a new EP tube and vacuum-concentrated to dryness at 37°C. The dried residues were then reconstituted in 150 uL of chloroform: methanol (3:1) and centrifuged at 16,000 *g* at 4°C for 15 minutes. The supernatant (100 µL) was analyzed by liquid chromatography tandem mass spectrometry (LC-MS/MS). Quality control (QC) samples were prepared by mixing equal volumes of more than half of the samples to be tested, with each QC sample volume equal to that of the samples under examination. For plasma samples: 300 µL of pre-cooled chloroform: methanol (3:1) was added to 100 µL of plasma sample. After centrifuging at 16,000 *g* at 4° for 15 minutes, the lower organic phase was collected and then vacuum-concentrated. The extracts were then reconstituted with 350 uL of chloroform: methanol (3:1), and centrifuged at 16,000 *g* at 4° for 15 minutes. The supernatant (300 µL) was analyzed by LC-MS/MS.

### Lipidomics data collection and analysis

The reconstituted samples were loaded for LC-MS/MS analysis in a randomized order using the UltiMate 3000 UHPLC System coupled with the Orbitrap Exploris 480 high-resolution mass spectrometer (Thermo Fisher Scientific). The lipidomics solution was eluted from a C18 analytical column (2.1 × 50 mm, 1.8 µm, Part number 186003535, Waters) with a VanGuardTM Pre-Column 2.1 × 5 mm, 1.8 µm (Part number1186003976, Waters) using a 22-min elution gradient: 0–3 min, 30%–35% of phase A; 3–5 minutes: 35%–65% phase A; 5–14 minutes: 65%–98% phase A; 14–18 minutes: 98% phase A; 18.1–22 minutes: 30% phase A. Mobile phase A was a mixture of acetonitrile and water (6:4 v/v) with 10 mM ammonium acetate and 0.1% acetic acid, and phase B was isopropanol and water (9:1 vol/vol) with the same additives. The flow rate was 0.35 mL/min, the column oven temperature was maintained at 55°C, and the injection volume was 1 µL. Positive and negative ion mass spectrometry data were acquired using a HESI source at +3,500V and −3,500V spray voltages, respectively. Data were acquired in the data-dependent acquisition (DDA) mode with a full scan covering *m/z* 150 to 2,000 at a resolution of 60,000 and MS/MS scan at 15,000 (max injection time was 22 ms) for top five precursor ions. Dynamic exclusion was performed for 10 seconds. The final data sets were analyzed using the LipidSearch (Version 5.0, Thermo Fisher Scientific) against the software-embodied database containing >1.5 million lipid ions and their predicted fragment ions. Peak detection was performed using the following parameters: bunching factor MS1 and MSn, 3; MS1 intensity threshold, 30,000; same peak RT threshold, 0.01; quant intensity ratio threshold, 3; baseline to max ratio threshold, 1; max peak charge, 2; charge detect minimum peak number, 2. Lipid molecular species were identified using a precursor ion mass tolerance of 10 ppm; a product ion mass tolerance of 10 ppm, and a product ion intensity threshold of 1.0%. Peak alignment was performed as follows: Calc. Method, Max; Filter Type, Setting Filter; RT Tolerance, 0.25 minutes; RT Correction Tolerance, 0.5 minutes; Intensity Ratio Threshold, 1.5; Valid Peak Rate Threshold, 0.5; m-Score, 5.0.

### Fecal proteomic sample preparation

About 750 µL of protein lysis buffer (10 mM tris(2-carboxyethyl) phosphine (TCEP, Sigma), 6M guanidinium hydrochloride (GdmCI, Sigma), and 40 mM chloroacetamide (CAA, Sigma) in 100 mM Tris buffer) were added to 150 mg of the fecal sample, vortexed for 60 seconds, and then centrifuged at 16,000 *g* at 4°C for 15 minutes. The supernatant was mixed with 125 µL trichloroacetic acid, vortexed for 1 minute, and incubated at 4°C for 1 hour. After being centrifuged at 16,000 *g* at 4°C for 15 minutes, the pellets were washed three times with ice-cold acetone. After impurity removal with acetone, 20 µg of proteins was digested with 0.2 µg of trypsin (final concentration 1% wt/wt) and then desalted and cleaned with Evotip.

### Fecal proteomic data acquisition and analysis

An Evosep One (Evosep Biosystems) was used as the liquid separation system, analyzing 30 samples daily with standardized gradients (SPD30). For enhanced sensitivity and accuracy in sample analysis, disposable trapping columns with C18 filling were used for sample pretreatment. Nano-RPLC with 1.9 µm C18 beads filled in a 15 cm ×150 µm capillary column was used for chromatographic separation. Mobile phase A was consisted of 99.9% water and 0.1% formic acid, and phase B consisted of 99.9% acetonitrile and 0.1% formic acid. Data were detected in the positive ion mode using an Orbitrap Fusion Lumos Tribrid mass spectrometer equipped with a Nanospray Flex ion source, with a spray voltage of 2,200 V, and data were acquired in the ata-Dependent Acquisition (DDA) mode with ion trap fragmentation.

Protein identification was performed using the search engine PEAKS against a protein database, containing 130,975,891 sequences from human, microbial, and dietary organisms (14). The mass tolerance of precursor and fragment ions was set to 15 ppm and 0.03 Da, respectively. Trypsin was set to be the cleavage enzyme, allowing a maximum of three missing cleavage sites. Carbamidomethylation of Cys, acetylation of protein N-terminus, Met oxidation, Asn and Gln deamidation, and Pyro-glu from Gln were specified as variable modifications, with a maximum of three per peptide. All proteins identified in the first step without false discovery rate (FDR) control were used for the second step search with a false discovery rate (FDR) set to 1% at both protein and peptide levels using Maxquant for label-free protein quantification.

### Statistical analysis

All count data were represented as frequency, and χ tests were used for statistical testing. Prior to any statistical analysis, the peak intensities of all detected proteins and lipids were normalized and scaled by sum and Pareto scaling, respectively. All measurement data (continuous variables) were represented as mean ± standard deviation. If the data followed a normal distribution and had equal variance, Student’s *t*-test was used. For rank data or unequal variances, the Wilcoxon rank-sum test was used, and the *P*-values from multiple comparisons were corrected for false discovery rate (FDR). All statistical analyses were two-sided, with *P* < 0.05 considered statistically significant. Omics data processing: original quantitative data matrices from different omics were imported into MetaboAnalyst (https://www.metaboanalyst.ca/) for further processing and analysis: data filtering through interquartile range (IQR) to remove baseline noise; pareto scaling for data normalization; principal component analysis (PCA) and orthogonal partial least squares-discriminant analysis (OPLS-DA) for unsupervised multivariate data analysis, with VIP values as one of the differential lipid screening indicators. Spearman correlation analysis was conducted for intergroup association analysis. Pathway analysis was performed using the Kyoto Encyclopedia of Genes and Genomes (KEGG) to identify relevant metabolic pathways. Multiple comparisons were adjusted using Benjamini–Hochberg test with significance threshold BH-adjust *P* < 0.05 and fold-change >1.5. Volcano plots and heatmaps were analyzed and plotted using R software (version 4.2.0), and the intergroup association analysis network map was visualized with Cytoscape (version 3.9.1).

## RESULTS

This study included 33 healthy individuals and 30 patients diagnosed with AMI. The two groups were well-matched in terms of sex, age, body mass index, etc. (*P* > 0.05). However, significant differences appeared in biochemical parameters, such as levels of triglycerides, lactate dehydrogenase, creatine kinase, creatine kinase isoenzyme-MB, cardiac troponin I, and B-type natriuretic peptide (*P* < 0.05), though no significant differences were observed in total cholesterol, high-density lipoprotein cholesterol, or low-density lipoprotein cholesterol level (*P* > 0.05) (Table S1).

### Overview of host and microbial lipidome and proteome profiling in AMI patients

**Fig 1:**
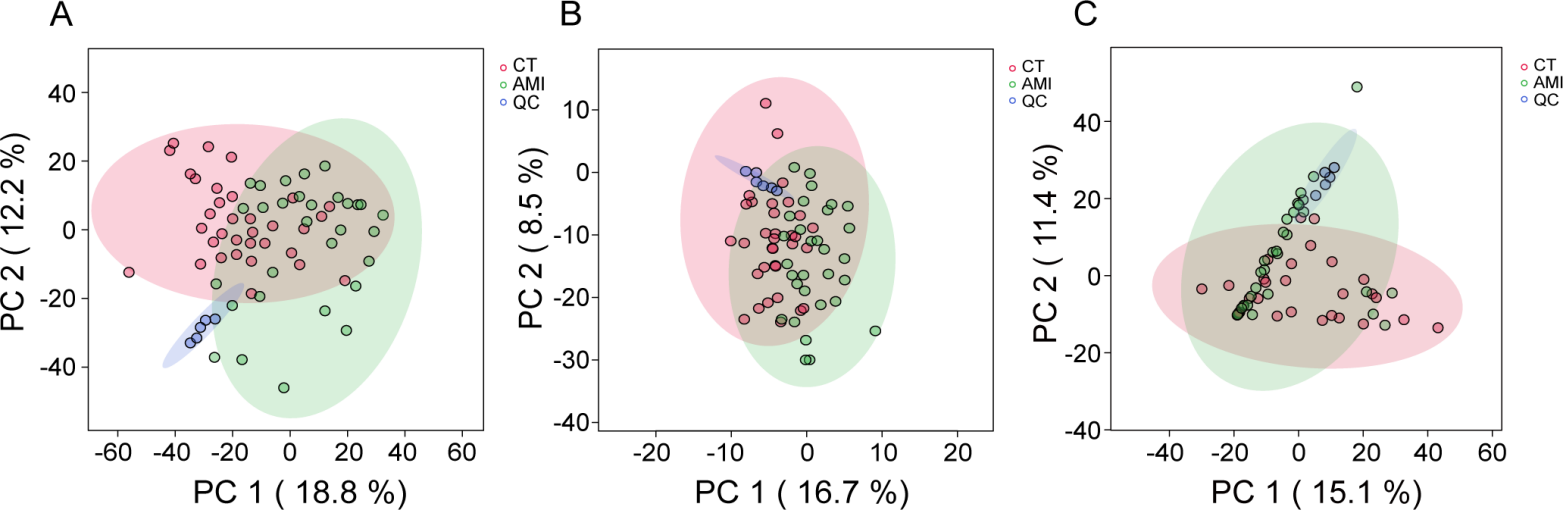
Quality control of mass spectrometry data. (**A**) Principal component analysis (PCA) of (**A**) the gut lipidomics data, (**B**) plasma lipidomics data, and (**C**) metaproteomics data.

To validate the reliability of LC–MS/MS detection, PCA was performed on gut lipidomics, plasma lipidomics, and gut metaproteomics data. There was a distinct separation between the AMI and control groups across all three omics data sets (Fig. 1). The QC samples clustered tightly, reflecting robust experimental conditions and stable system performance during mass spectrometry detection, which ensured reproducible results.

### Identification of fecal lipids and potential biomarkers

Through meticulous analysis of fragment ions and related acyl chain fragments, we identified a comprehensive profile of 3,919 specific lipids in the feces, spanning seven lipid categories. Glycerolipids were predominant, contributing 44.71% (1,754) to fecal lipids, whereas prenol lipids were the least abundant, accounting for only 0.1%.

**Fig 2:**
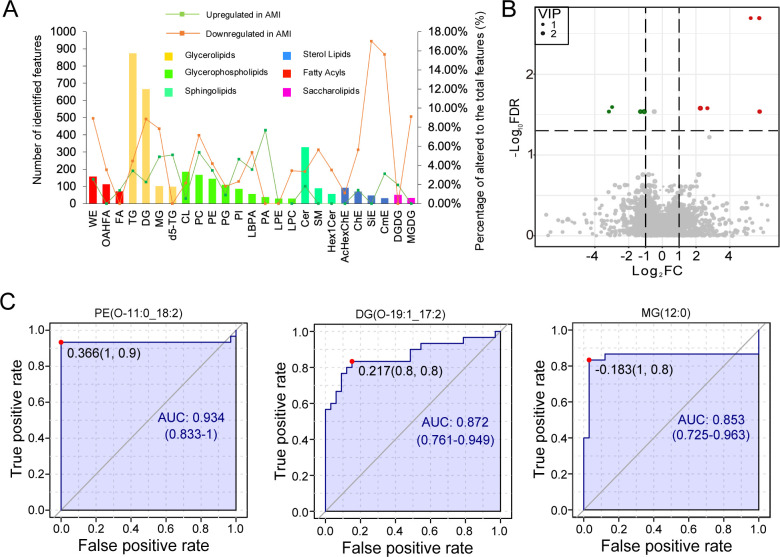
Classification, differential analysis, and molecular marker screening of gut lipids. (**A**) Differential distribution of the top 25 lipid subclasses in acute myocardial infarction (AMI). (**B**) Volcano plot for differential analysis of gut lipid molecules. (**C**) Receiver operating characteristic (ROC) curve analysis of three significantly different gut lipids including.

We compared the top 25 fecal lipid subclasses to elucidate the differences in the distribution between the AMI and control groups. In comparison to the control group, four and two phosphatidylinositol lipid species levels were higher and lower in the AMI group (FDR < 0.05), representing 4.65% and 2.33% of total phosphatidylinositol lipids, respectively. In addition, three phosphatidic acid species were elevated in the AMI group (FDR < 0.05) (Fig. 2A).

In combination with the Wilcoxon rank-sum test and multivariate analysis-based variable importance in projection (VIP) values, differentially regulated fecal lipids were identified between the AMI and control groups (VIP >1, FDR < 0.05, fold-change FC ≥2 or ≤0.5). In detail, PI (14:1_3:0), CerPE (d16:0_17:0), MG (12:0), DG (O-19:1_17:2), and TG (8:0_10:0_12:0) were upregulated, while PE (O-11:0_18:2), PI (31:6), TG (29:8_18:1_30:8), and TG (55:1_18:0) were downregulated in AMI patients (Fig. 2B, Table S2). The diagnostic potential of these nine significantly regulated lipids was assessed using areas under the curve (AUC) values, all of which were significant (*P* < 0.05) (Table S3). Among them, PE (O-11:0_18:2) exhibited the highest AUC value (0.934), followed by DG (O-19:1_17:2) and MG (12:0) with AUC values of 0.872 and 0.853, respectively, indicating their strong diagnostic potential as biomarkers for AMI (Fig. 2C).

### Plasma lipid identification and potential biomarker screening

In plasma, we detected 839 lipid species, classified into 30 subclasses and six major classes. Glycerophospholipids were the most abundant, accounting for 44.66% of the total plasma lipids (375 in 839), whereas sterols were the least represented, accounting for only 0.12%.

**Fig 3:**
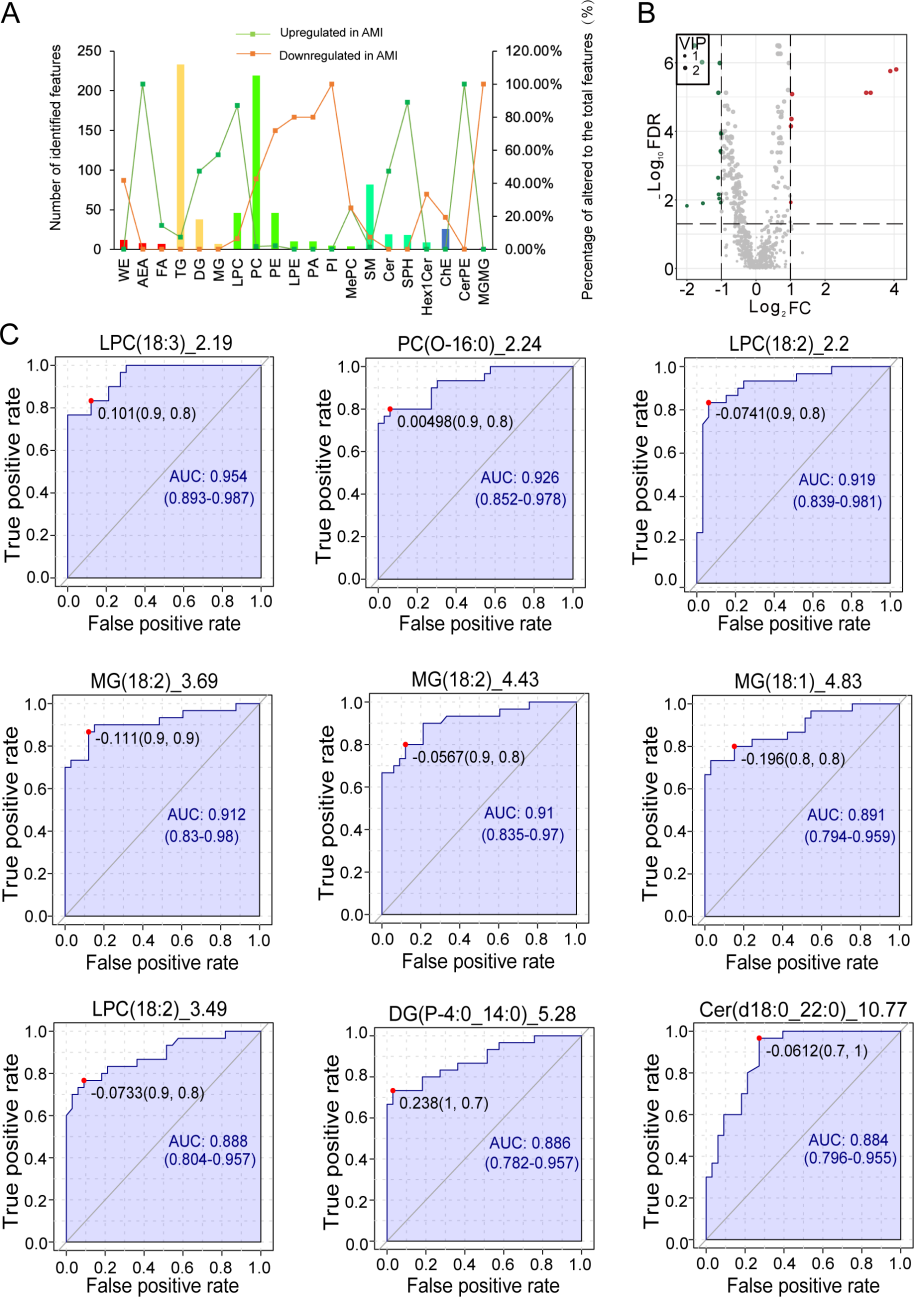
Classification, differential analysis, and molecular marker screening of plasma lipids. (**A**) Differential distribution of the top 20 lipid subclasses in acute myocardial infarction (AMI). (**B**) Volcano plot of the differentially expressed plasma lipid species. (**C**) Receiver operating characteristic (ROC) curves of the nine significantly differentially expressed plasma lipid species.

We compared the top 20 identified plasma lipid subclasses to elucidate the differences in distribution between the AMI and control groups. The ratio of upregulated glycerophospholipids was lower than that of the downregulated ones in the AMI group. For instance, phosphatidylethanolamines (PEs) were upregulated by 2.17% and downregulated by 71.74%; lyso-phosphatidylethanolamines (LPEs) and phosphatidic acids (PAs) were both downregulated by 80%, with none upregulated; phosphatidylinositols (PIs) were downregulated by 100%. In terms of phosphatidylcholines (PCs), 1.83% (four in 219) were elevated and 42.47% (93 in 219) were reduced in the AMI group (FDR < 0.05). (Fig. 3A).

Eight upregulated and 15 downregulated plasma lipids in AMI patients were identified by differential analysis (Fig. 3B; Table S4). Interestingly, the upregulated lipids were sphingolipids (Cer, SPH) and glycerolipids (DG, MG). In contrast, the downregulated lipids were mainly glycerophospholipids (PI, PC, LPC, PE, and PA) with only one exception, AcCa(2:0)_0.4. ROC curve analysis identified nine species with AUC values > 0.85 (*P* < 0.05), with LPC(18:3)_2.19 exhibiting the highest AUC value (0.954), followed by PC(O-16:0)_2.24 and LPC(18:2)_2.2 (AUC, 0.926 and 0.919) (Fig. 3C; Table S5).

### Identification of the fecal microbiota via fecal metaproteomics

**Fig 4:**
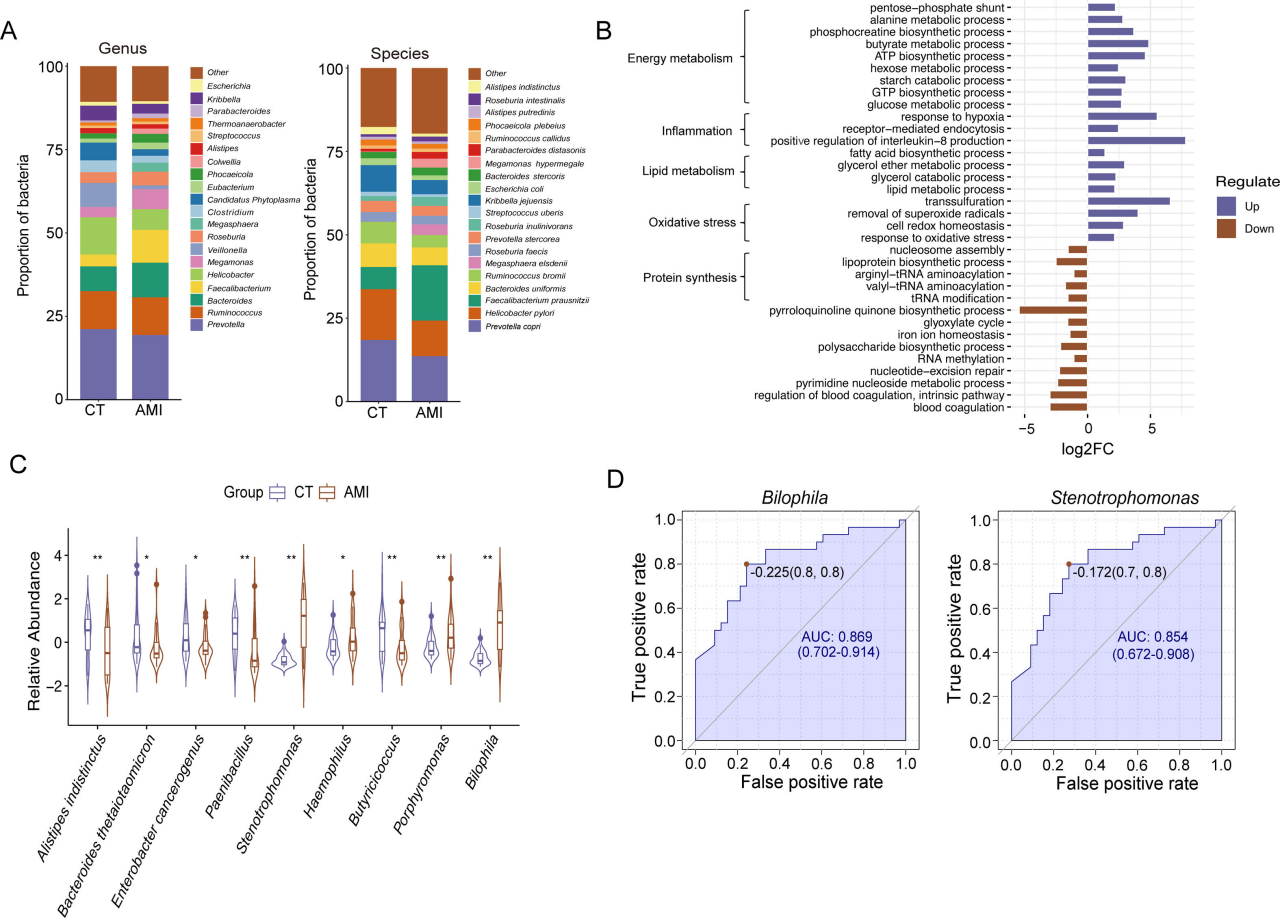
Identification, differential analysis, and biomarker screening of gut microbiota. (**A**) Relative abundances of the major bacterial genera in the acute myocardial infarction (AMI) and control groups. (**B**) Biological processes of the representative microbial proteins. (**C**) Violin plot of differences in relative abundance at genus and species levels between the AMI and control groups. (**D**) Receiver operating characteristic (ROC) curves of the significantly differentially abundant gut microbiota.

Leveraging the advantage of metaproteomics, we annotated 498 microbial species within fecal samples. Among which, *Prevotella copri*, *Escherichia coli*, *Helicobacter pylori*, *Bacteroides uniformis*, and *Faecalibacterium prausnitzii* comprise over 50% of the total abundance (Fig. 4A). Nine bacterial species exhibited significant differences in relative abundance at the genus and species levels between the control and AMI groups (FDR < 0.05) (Fig. 4C; Table S6). Species with a lower abundance in the AMI group were predominantly from the Firmicutes, whereas those with a higher abundance were mainly from Proteobacteria and Bacteroidetes. To assess the biomarker potential of these differentially abundant gut microbes, we calculated their AUC values (Table S7). *Bilophila* and *Stenotrophomonas* exhibited the highest AUC values of 0.869 and 0.854, respectively, indicating their capability for predicting AMI (Fig. 4D).

Gene Ontology (GO) analysis of microbial protein function identified 117 biological processes that were significantly enriched in the AMI group in comparison to controls (FDR < 0.05) (Fig. 4B, Table S8). Oxidative stress, lipid metabolism, inflammatory responses, and energy metabolism were significantly enriched in the AMI group. Specifically, lipid metabolic processes such as fatty acid biosynthesis, glycerol ether metabolism, glycerol catabolism, and overall lipid metabolism were notably enriched.

### Host protein identification and biological function analysis

**Fig 5:**
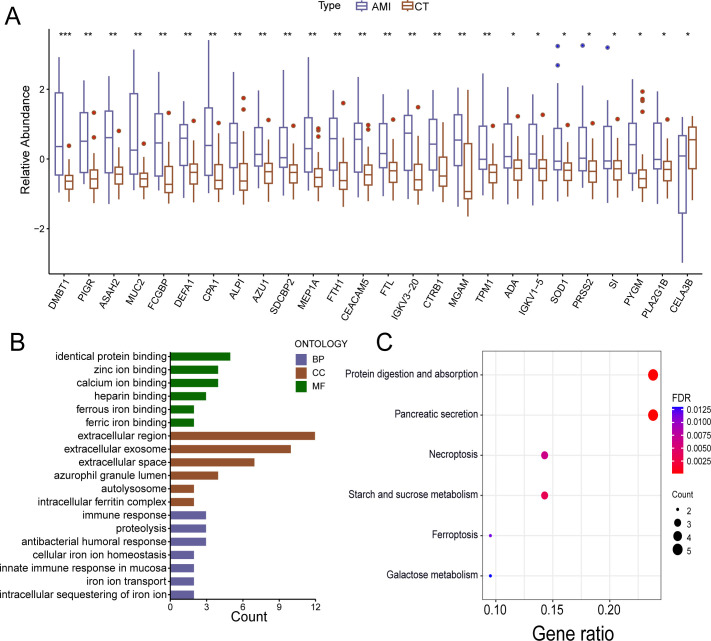
Relative abundances of the representative gut host proteins, gene ontology (GO), and Kyoto Encyclopedia of Genes and Genomes (KEGG) enrichment. (**A**) Differential analysis of host proteins in the acute myocardial infarction (AMI) and control groups. (**B**) GO and (**C**) KEGG enrichment analysis of differentially expressed host proteins. * FDR < 0.05, ** FDR < 0.01, and *** FDR < 0.001.

Using an LC-MS/MS-based untargeted proteomics approach, 148 host proteins were identified, accounting for 4.57% of the gut protein profile. Among these, 26 host proteins exhibited significant differential expression between the two groups (FDR < 0.05). Except for CELA3B, which was downregulated, the remaining 25 proteins were upregulated in the AMI patients (Fig. 5A).

Functional enrichment analysis revealed that the differentially expressed host proteins were primarily involved in the antibacterial humoral response, intracellular sequestration of iron ions, iron-ion transport, the innate immune response in the mucosa, proteolysis, cellular iron ion homeostasis, and the immune response (Fig. 5B). KEGG pathway analysis revealed that the most enriched pathways were the protein digestion and absorption pathway and the pancreatic secretion pathway, followed by the necroptosis pathway and the starch and sucrose metabolism pathway. The ferroptosis pathway and galactose metabolism pathway were also enriched in the AMI group (Fig. 5C).

### Correlation analysis between fecal microbiota and lipids

**Fig 6:**
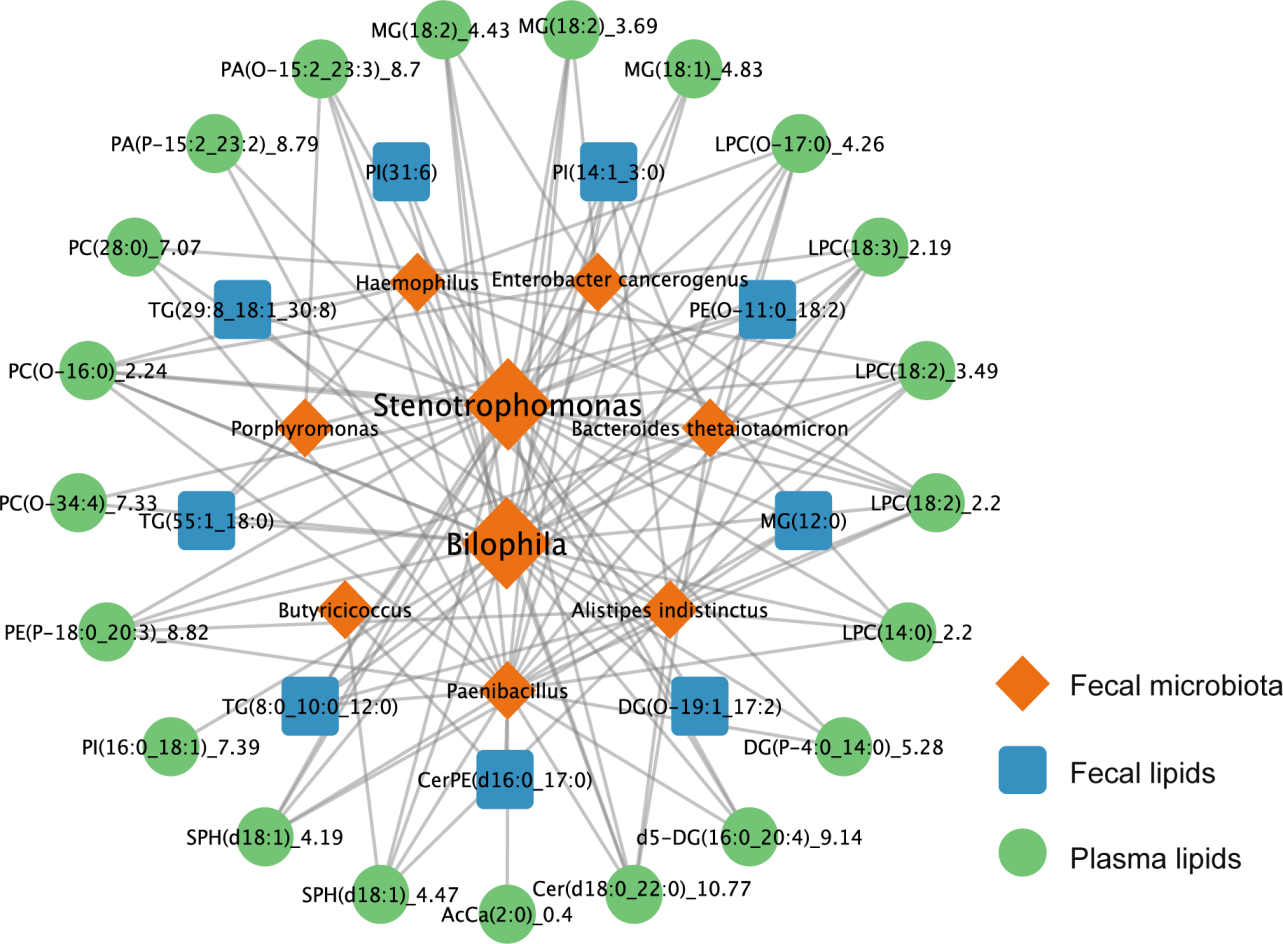
Correlation analysis between the differentially regulated gut microbiota and differentially regulated gut and plasma lipids.

Correlation analysis revealed a close association between gut microbiota abundances and lipid levels in both fecal and plasma samples of AMI and control groups. The abundances of *Stenotrophomonas* and *Bilophila* were notably correlated with lipid levels. Specifically, *Stenotrophomonas* was positively correlated with levels of SPH (d18:1)_4.47 (*r* = 0.52, FDR = 1.7e-04) and negatively correlated with those of LPC (18:2)_2.2 (*r* = −0.42, FDR = 6.4e-04), and phosphatidylcholine (PC; O-16:0)_2.24 (*r* = −0.44, FDR = 3.2e-03), whereas *Bilophila* was positively correlated with ceramide (Cer; d18:0_22:0)_10.77 levels (*r* = 0.51, FDR = 2.1e-03) and negatively correlated with LPC (18:2)_3.49 (*r* = −0.42, FDR = 6.7e-03) and LPC (18:2)_2.2 (*r* = −0.47, FDR = 1.3e-03) levels (Fig. 6). This suggests that interactions between *Bilophila* and *Stenotrophomonas* and fecal and plasma lipids may play important roles in lipid metabolism associated with AMI pathogenesis.

### Correlation analysis between gut microbiota abundances and clinical indicators

**Fig 7:**
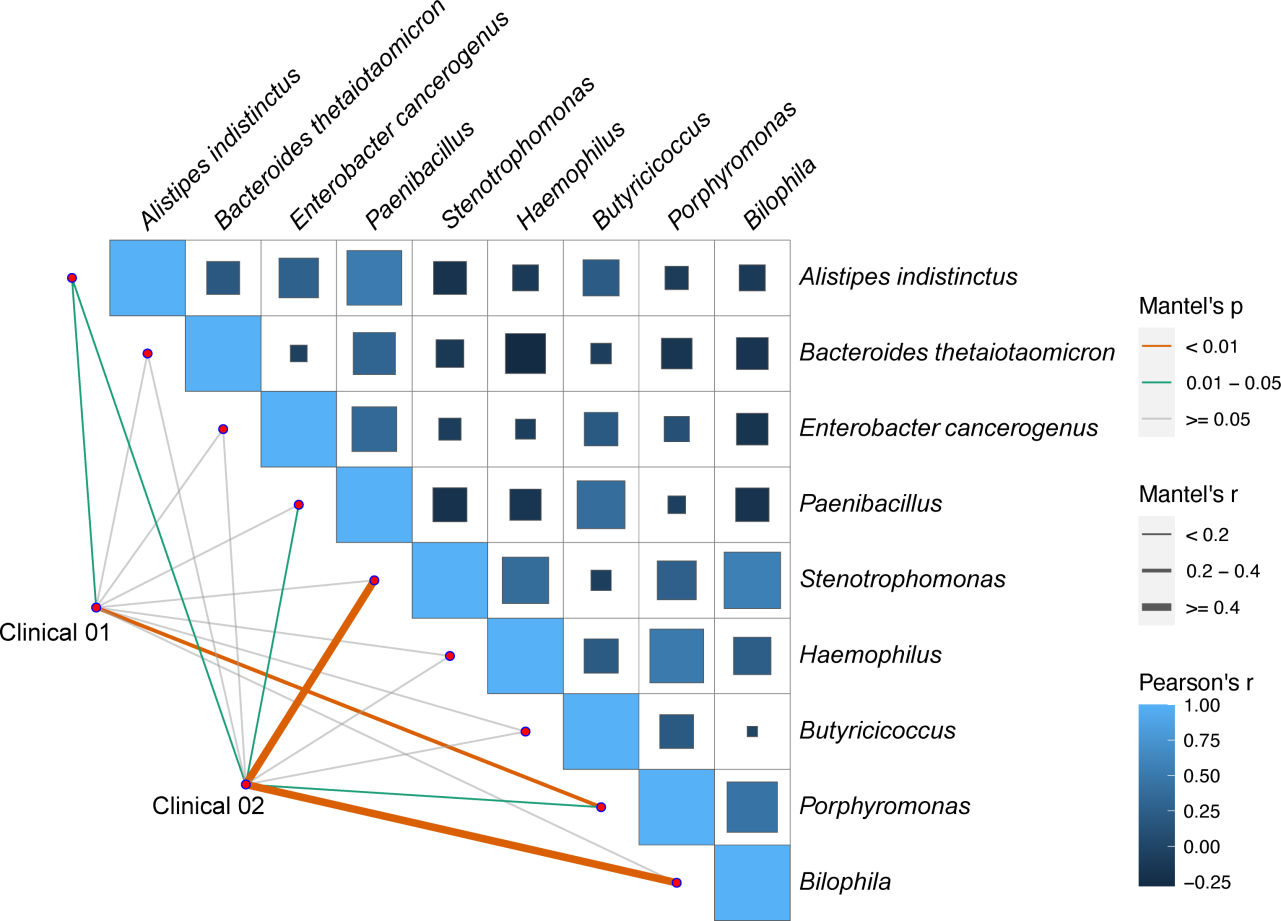
Mantel test analysis of the association between the gut microbiota, blood lipids, and myocardial injury clinical indicators.

To further clarify the role of the gut microbiota in cardiovascular health, we performed Mantel test correlation analysis between the differentially abundant gut microbes and clinical blood lipid and myocardial injury indicators in the AMI group. *Alistipes indistinctus* and *Porphyromonas* abundances significantly correlated to Clinical 01 blood lipid indicator levels (TC, LDL-C; Mantel’s *r* > 0.2, Mantel’s *P* < 0.05). Additionally, *A. indistinctus*, *Bilophila*, and *Stenotrophomonas* were closely related to Clinical 02 myocardial injury indicators (cTnI, CK, CK-MB, LDH, and BNP; Mantel’s *r* > 0.2, Mantel’s *P* < 0.05) (Fig. 7). These results suggest that *A. indistinctus* and *Porphyromonas* may influence plasma lipid metabolism in the early stages of AMI, whereas *Bilophila* and *Stenotrophomonas* may participate in both pre-and post-AMI, affecting the increase in myocardial injury-related indicators.

## DISCUSSION

The present study applied a multi-omics approach to identify changes in lipid metabolism in patients with AMI, elucidating the association between lipids and the gut microbiota and their roles in the occurrence and development of AMI. This is the first application of gut lipidomics and gut metaproteomics in a combined multi-omics analysis of the biomarkers and pathological mechanisms of AMI. Here, we identified a relationship between plasma levels of atherosclerosis-related lipids and AMI, revealing novel therapeutic targets for AMI and highlighting the importance of lipid management in the prevention of cardiovascular disease.

The gut is a key site for lipid metabolism. Abnormal gut lipid metabolism is closely related to the development of diseases such as ulcerative colitis and metabolic syndrome (21), although the relationship between gut lipid metabolism and AMI remains unclear. In a large prospective epidemiological study using nontargeted metabolomics and involving 1,028 participants, four lipid species were identified as independent risk factors for coronary heart disease, namely, LPC (18:1), LPC (18:2), MG (18:2), and SPH (28:1) (22). In this study, ROC analysis of differentially expressed gut and plasma lipids identified potential biomarkers for AMI. Specifically, three gut lipid species and nine plasma lipid species exhibited AUC values greater than 0.85, indicating that these lipid molecules may serve as potential clinical diagnostic biomarkers for AMI.

Recently, the role of the gut microbiota in the occurrence and development of AMI and its potential value in the clinical diagnosis and treatment of AMI have attracted increasing interest. Here, we used gut metaproteomics to explore differences in the gut microbiota between patients with AMI and healthy individuals. Metaproteomics can reveal functional features related to the potential physiological state of the body and can provide insights into the interconnections between microbial diversity, function, and their impact on host biology (23, 24), offering a reference for the early diagnosis and treatment of diseases. Here, nine gut microbial species exhibited significantly different abundances between the AMI and control groups. Two of these, in the genera *Bilophila* and *Stenotrophomonas*, had AUC values > 0.85, with AUCs of 0.869 and 0.854, respectively. These findings reveal that ROC classification performed well overall, accurately distinguishing between patients with AMI and healthy controls. Therefore, *Bilophila* and *Stenotrophomonas* can be considered potential biomarkers for the early diagnosis of AMI, providing a potential noninvasive method for diagnosing AMI.

The relationship between the gut microbiota and lipid metabolism has generated great interest. The gut microbiota can affect the lipid metabolism of the host, and abnormalities in lipid metabolism may lead to changes in the composition of the gut microbiome (25). Elucidating this association between lipid metabolism and the gut microbiota in acute myocardial infarction may provide new perspectives on the pathophysiological mechanisms of acute myocardial infarction. Our findings indicate a significant correlation between gut microbiota abundance and both gut and plasma lipid levels in patients with AMI, and particularly between *Bilophila* and *Stenotrophomonas* abundance and lipid metabolism.

*Bilophila* abundance and triglyceride (55:1–18:0) levels were significantly positively correlated (*r* = 0.49, FDR = 5.6e-04). Gut lipid triglycerides, which are synthesized from fatty acids in the gut, are absorbed into the bloodstream and metabolized in the liver. High concentrations of gut triglycerides could lead to lipid deposition within vessels, forming atherosclerotic plaques, and increasing the risk of AMI (26). Gut triglyceride levels can affect the number and size of low-density lipoprotein particles in the blood, thereby increasing the risk of thrombus formation (27). Triglycerides may exacerbate oxidative stress and inflammatory responses, causing damage to the gut mucosal barrier, allowing gut bacteria and toxins to enter the bloodstream (28). Therefore, we hypothesized that interactions between *Bilophila* and gut triglycerides play a crucial role in regulating gut mucosal inflammatory responses and maintaining the stability of the gut mucosal barrier.

*Bilophila* abundance was strongly positively correlated with plasma sphingolipid Cer (d18:0_22:0)_10.77 levels (*r* = 0.51, FDR = 2.1e-03). Ceramide, an important sphingolipid, plays key roles in many biological processes, including apoptosis, autophagy, inflammation, fatty acid oxidation, aging, and endoplasmic reticulum stress (25). *Bilophila* can metabolize compounds such as sulfates in the gut and produce metabolites such as thiols, which can cause an inflammatory response in the gut mucosa (29). Thus, we speculate that *Bilophila* may trigger an inflammatory response in the gut mucosa, disrupting gut mucosal barrier integrity and allowing lipids such as ceramides and other harmful substances to enter the bloodstream. Once in the circulation, these substances trigger a systemic inflammatory response, thereby increasing the risk of AMI.

This study has a few limitations. It employed a cross-sectional research design, which can only observe phenomena at a specific point in time and may not fully capture the dynamic interactions between gut microbiota and lipid metabolism. Additionally, the small sample size limits the generalizability and validity of the findings. Future research should combine longitudinal studies with cross-sectional research to better track dynamic changes and elucidate the interrelationships between gut microbiota and lipid metabolism, as well as their impacts on AMI development. Further studies with larger sample sizes are essential to enhance the generalizability and validity of the findings.

### Conclusions

This study provides a novel perspective on the associations between lipid metabolism, the gut microbiota, and AMI. The potential biomarkers identified here hold promise for enhancing AMI diagnosis and prognosis. This work provides insight into the role of the gut microbiota in lipid metabolism, offering new avenues for understanding and managing AMI. These findings contribute to advancing our knowledge of AMI pathogenesis, with the potential to improve the early clinical diagnosis and treatment of this life-threatening condition.

## SUPPLEMENTAL MATERIAL

Table S1 spectrum.02370-24-s0001.csv Comparison of general data and clinical indicators between AMI group and CT group.

Table S2 spectrum.02370-24-s0002.csv Statistical table for analysis of differences in intestinal lipid molecules.

Table S3 spectrum.02370-24-s0003.csv AUC of 9 significantly different intestinal lipids.

Table S4 spectrum.02370-24-s0004.csv Statistical table for analysis of differences in plasma lipid molecules.

Table S5 spectrum.02370-24-s0005.csv AUC of the first 9 significant differences in plasma lipids.

Table S6 spectrum.02370-24-s0006.csv Differential strains between AMI group and CT group.

Table S7 spectrum.02370-24-s0007.csv AUC of significantly different intestinal microorganisms.

Table S8 spectrum.02370-24-s0008.csv Biological process of representative microbial proteins.

ASM does not own the copyrights to Supplemental Material that may be linked to, or accessed through, an article. The authors have granted ASM a non-exclusive, world-wide license to publish the Supplemental Material files. Please contact the corresponding author directly for reuse.
